# Dietary and plasma blood α-linolenic acid as modulators of fat oxidation and predictors of aerobic performance

**DOI:** 10.1186/s12970-020-00385-2

**Published:** 2020-11-16

**Authors:** Aleksandra Y. Lyudinina, Ekaterina A. Bushmanova, Nina G. Varlamova, Evgeny R. Bojko

**Affiliations:** Department of Ecological and Medical Physiology, Institute of Physiology, Ural Branch, Russian Academy of Sciences, Pervomaiskaya str. 50, 167982 Syktyvkar, Russia

**Keywords:** Maximal fat oxidation, N-3 PUFAs, α-Linolenic acid, Aerobic performance, Skiers

## Abstract

**Background:**

Among n-3 polyunsaturated fatty acids (PUFAs), the most important is α-linolenic acid (ALA). The biological activity of ALA is not equivalent to that of the long-chain n-3 PUFAs, and it has pleiotropic effects, such as functioning as an energy substrate during long-term training when carbohydrate reserves are depleted. The purpose of this investigation was to study the link between the essential dietary and plasma ALA and aerobic performance, which is estimated via maximal fat oxidation (MFO), among skiers.

**Methods:**

Twenty-four highly trained male athletes from the Russian cross-country skiing team participated in the study. ALA intake was determined by an original program used to assess the actual amount and frequency of fat consumption. The plasma level of ALA was determined using gas-liquid chromatography. The skiers’ aerobic performance was estimated via MFO and determined by indirect calorimetry using the system “Oxycon Pro”.

**Results:**

The consumption of ALA in the diet in half of the skiers was below the recommended level at 0.5 ± 0.2 g/day. The deficiency of plasma ALA levels was on average 0.2 ± 0.1 Mol% for almost all participants. The consumption of ALA in the diet and its level in plasma were associated with MFO (r_s_ = 0.507, *p* = 0.011; r_s_ = 0.460, *p* = 0.023). Levels of ALA in plasma (*p* = 0.0523) and the consumption of ALA in the diet (*p* = 0.0039) were associated with high aerobic performance.

**Conclusions:**

ALA in the diet of the athletes may be used as nutritional support to increase MFO and aerobic performance.

## Background

High aerobic potential is crucial in top-performance sports, such as skiing [1, 2], and endurance capacity is highly related to maximal cardiac output and the ability of skeletal muscle to oxidize fat and carbohydrates [3]. Important metabolic training adaptations include increased glycogen stores and increased fat oxidation capacity, two parameters that are crucial for athletes competing in events lasting more than 2–3 h [3]. Strategies for increasing glycogen stores are fairly well characterized, whereas strategies for developing higher fat oxidation rates have recently received increasing interest by scientists, coaches and athletes [3–6]. According to the literature [3, 7–10], high aerobic training in endurance sports is associated with increased utilization of body fat.

Assessments of the functional state of highly trained athletes take into account the rate of oxidation of energy substrates, i.e., carbohydrates and fats [11]. The use of fats as a source of energy depends on the type of sport; the training status, body fat, age, sex, and glycogen content in the muscles of the athlete; and the intensity and duration of the physical activity [8, 9]. The role of nutritional factors in improving maximal fat oxidation (MFO) and endurance of athletes in the preparatory and competitive periods and during rehabilitation after intense exercise is poorly understood [3, 5, 6].

The association between essential nutrients (e.g., vitamins) and physical efficiency in athletes has been well studied and demonstrated [1, 12]. The study of essential components of nutrition, particularly n-3 polyunsaturated fatty acids (PUFAs), is of great interest in sports worldwide due to their significant role in improving physical performance [13–15]. n-3 PUFAs are necessary for the energy supply for muscle activity and contribute to maximal oxygen uptake (VO_2_max) [16] and performance improvement in cyclical sports athletes [14].

Skeletal muscle lipid composition is sensitive to dietary changes, thus indicating the potential for nutritional interventions to influence skeletal muscle metabolic processes. The essential fatty acid (FA) α-linolenic acid (ALA) can function as an energy substrate during long-term training when carbohydrate reserves are depleted [17, 18]. However, research has not focused on the relationship between the consumption of ALA and its content in the blood based on MFO and the aerobic performance of highly trained athletes.

The purpose of this investigation was to study the link between the essential dietary and plasma ALA levels and the aerobic performance of skiers estimated via MFO.

## Materials and methods

### Participants

We studied members of the national skiing team during the general training season. Thirty-three participants were initially enrolled in this study. However, only 24 highly trained male cross-country skiers (mean ± SD: age 22.8 ± 4.2 years, body height 178.0 ± 4.2 cm, body mass 178.0 ± 4.2 kg and maximal oxygen uptake 59.7 ± 6.9 mL·min^− 1^·kg^− 1^, body fat 9.5%) completed the entire study protocol and were included in the analyses. The anthropometric and physiological parameters of the examined athletes are shown in Table 1.

**Table 1:** Anthropometric characteristics

Characteristics	M ± SD
Age, years	22.8 ± 4.2
Body mass, kg	72.7 ± 4.6
Body height, cm	178.0 ± 4.2
Fat mass, %	10.5 ± 4.9
Body mass index, kg/m^2^	23.0 ± 0.1
VO_2_max, mL·min^− 1^·kg^− 1^	59.7 ± 6.9

*Note: Values are expressed as the mean (M) ± standard deviation (SD)*

**Fig. 1:**
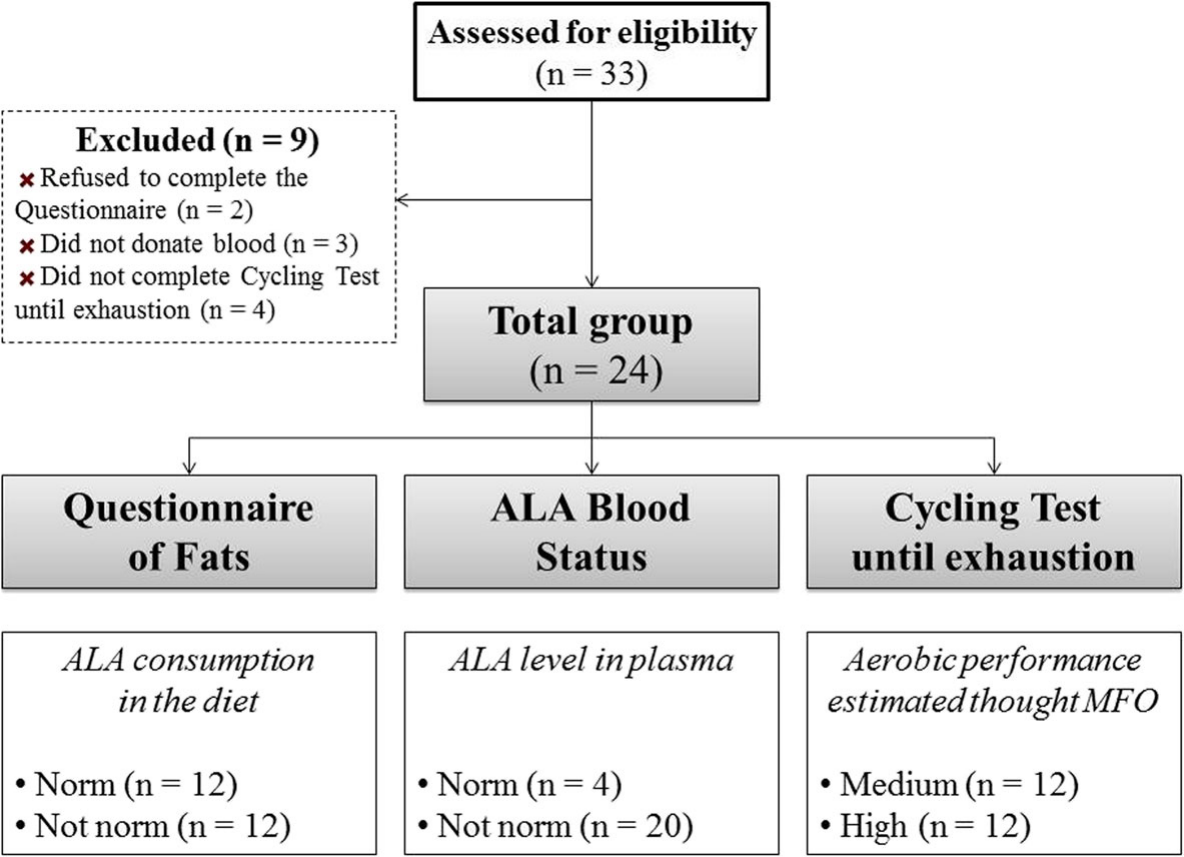
Flow chart of the study design

The criteria for qualifying for the study included age (18 to 33 years) and good health with a valid and up-to-date medical certificate confirming the athlete’s ability to practice sports. Exclusion criteria included illicit drug use, alcohol consumption greater than 1–2 drinks/week, special diet consumption within 3 weeks of the study’s commencement, history of acute and exacerbated chronic illnesses, signs of ARVI (Fig. 1).

The study protocol was reviewed and approved by the Local Research Bioethics Committee of the Institute of Physiology of the Komi Scientific Centre of the Ural Branch of the Russian Academy of Sciences. Written informed consent was obtained from all participants before the study began. All procedures were conducted in accordance with the ethical standards of the 1964 Helsinki Declaration [19].

### Experimental design

For part 1, highly trained male cross-country skiers met with the study assistant to receive detailed instructions for the completion of a modified 10-item Questionnaire of Products Containing Fat for one month (its description is presented below). ALA consumption in the diet was calculated based on the results of this Questionnaire of Fats (QFat).

For part 2, highly trained male cross-country skiers performed a cycling test until exhaustion and venous blood sampling was performed. The study procedure was again explained, and potential questions were answered before obtaining informed written consent. Venous blood samples of 9 mL were only taken before the cycling test until exhaustion and collected into EDTA-coated vacutainers. MFO was determined by indirect calorimetry with full gas Analysis. Data were collected during the competitive periods of 2017 and 2018.

### Questionnaire of fats (QFat)

The athletes were on a standardized balanced diet (in the SAF RK of the centre for sports training of national teams) and lived in the same city. The study was performed during the morning; therefore, all of the volunteers consumed a standardized meal (400–420 kcal) consisting of 78 En% (percentage of the total energy supplied by the entire meal) carbohydrates, 14 En% fat and 8 En% protein. The number of calories in the breakfast was not normalized according to body weight. The dietary intake of our participants was assessed using a questionnaire that included quantitative and frequency assessments.

An analogue of the QFat was a questionnaire based on the American n-3 PUFA FFQ by Sublette et al., which was developed and approved to estimate consumption of n-3 PUFAs in the New York area [20]. QFat is an on-line screening questionnaire developed by us independently and includes an assessment of the frequency consumption (for one month) of fat-containing products and the actual amount (in grams). The QFat was in the form used by the original programme “Assessing the adequacy of consumption of essential fatty acids” (certificate GR № 2,016,662,728 from 20.12.2016), on the basis of which the on-line service “Fatty Acids in Products” was created.

Participants completed a 10-item QFat that quantifies the intake of ALA. Briefly, the QFat enquires about the intake of foods, including dairy products, cheeses, vegetable oil, fat products, meat products, fish, sweets, nuts, and fast food. Special emphasis was placed on the ALA content of all products (e.g., vegetable oil, nuts, dairy products, meat products). For each food, participants reported the frequency of consumption over the past month, with responses ranging from never to several times each month, week, or day. If the participant responded that they consumed the food (any answer other than ‘never’), then they reported their typical portion size for each food.

Participants were asked to record all food consumed over a period of one month as precisely as possible. They were asked to indicate serving sizes using pictures from an adapted original nutritional programme “Fatty Acids in Products” developed by us. Whenever possible, individual ingredients were reported rather than final meals, including preparation methods, brand names, added oil, etc. The importance of not changing eating habits during the one month of recording was also stressed. Portion size responses were in units of grams for foods and oil.

### Cycling test until exhaustion

VO_2_max testing was performed on an ergometer bike (Ergoselect-100, Ergoline GmbH, Germany). The protocol included one minute of cycling without load (for adaptation) and then a stepwise load increase by 40 W in 2 min time increments starting with an initial load of 120 W. The pedalling speed was 60 rpm. Heart rate (HR) and work load were continuously recorded (every 15 s). Measurements (VO_2_ and VCO_2_) were taken throughout the exercise period using an automated gas analysis system (Jaeger Oxycon Pro, Wuerzberg, Germany) in “breath-by-breath” mode.

### “FATmax” test

To comprehensively define the relationship between the whole-body fat oxidation rate and exercise intensity, the “FATmax” test was used [21]. This graded exercise test elucidates whole-body fat oxidation rates across a range of exercise intensities as well as MFO and the intensity at which MFO occurs (FATmax) using indirect calorimetry with full gas analysis [8, 9, 22]. Calculations were performed using the original programme we developed (certificate GR № 2,019,613,060 from 06.03.2019). The test protocol is fat oxidation (g.min^− 1^) against VO_2_max during a graded cycling FATmax test, where MFO (g.min^− 1^), and FATmax indicate the intensity at which MFO occurs (W) [22]. According to the literature [8], aerobic performance, estimated through MFO, is divided into 3 groups: low (MFO < 0.37 g/min), medium (MFO 0.37–0.69 g/min) and high (MFO > 0.69 g/min). Normative values could be used to define the fat oxidation capacity of given research cohorts in exercise-metabolic studies in a manner analogous to VO_2_max based definitions of aerobic capacity [8].

### ALA blood status

The level of ALA in plasma was determined using gas-liquid chromatography. Blood samples were taken from fasting subjects at rest. Sample preparation included extracting lipids from plasma and obtaining FA methyl ethers using methanol and acetyl chloride as described by Lyudinina et al. [10]. Gas-liquid chromatography analysis of the FA methyl ethers was performed on a gas chromatograph (Crystal 2000М, Chromatek, Russia) with a flame ionization detector attached to a SUPELCOWAX (25 m × 0.23 mm) capillary column (Supelco, USA) at a temperature range of 170 °C to 250 °C (retention time, 2 min). The temperature increase rate was 4 °C/min (overall time, 25 min). Helium was used as the carrier gas, the volume rate was 0.6 ml/min, and the flow separation rate was 1/65. The evaporator temperature was 260 °C, and the detector temperature was 200 °C. FA identification was performed using Sigma standards. The quantitative analysis of the FА concentrations was performed using the internal standard of margarine acid solution (C17:0). The FA concentrations are expressed as a weight percentage of the total weight of FAs. The reported fasting means of plasma total FAs (mols%) in healthy adults were taken from a pre-existing source [23].

### Statistical analysis

The statistical procedures were performed and graphs were generated with Statistica software (version 8.0, StatSoft Inc., 2007, USA). The statistical results are expressed as the mean ± standard deviation (M ± SD). The significance of differences between the indicators was assessed using the nonparametric Mann–Whitney U-test and Friedman test (according to data normality). Data normality was verified with the Shapiro-Wilk test. The correlation coefficients between two variables were determined by Spearman’s rank analysis. A value of *p* < 0.05 was accepted as statistically significant.

## Results

The physiological parameters at the maximum ergometer bike level in our study are presented in Table 2. Our study showed a statistically significant increase in the indicators of the circulatory system at the peak of the load compared to that during rest before the test, which indicates the maximum intensity of the load.

**Table 2:** Physiological characteristics of the skiers during bicycle ergometer testing

Physiological parameters	Skiers	
	Steady state	Peak load
Heart rate, b/min	59.9 ± 8.6***	181.4 ± 14.2
Systolic blood pressure, mm Hg	112.5 ± 12.4***	190.8 ± 13.6
Diastolic blood pressure, mm Hg	77.1 ± 7.0	75.1 ± 15.3
Oxygen utilization quotient, ml/l	32.1 ± 3.5***	30.9 ± 6.2
Oxygen consumption, ml/min/kg	4.6 ± 0.8***	59.7 ± 6.9

Note: The values are presented as the M ± SD. The significant differences were determined by the Friedman criterion between the indicators at rest (*** p < 0.001) relative to the peak load

The HR at rest was 59.9 ± 8.6 beats/min, and the peak heart rate increased to 181.4 ± 14.2 beats/min (*p* = 0.00001). Systolic blood pressure (SBP) also significantly increased relative to that during the resting state (112.5 ± 12.4 mmHg) and was 190.8 ± 13.6 mmHg during the peak load (p = 0.00001). Diastolic blood pressure (DBP) did not change significantly and was 77.1 ± 7.0 mmHg at rest and 75.1 ± 15.3 mmHg at peak load (*p* = 0.393). VO_2_max during the peak load increased significantly and reached 59.7 ± 6.9 ml/min/kg (*p* = 0.003) (Table 2).

**Fig. 2:**
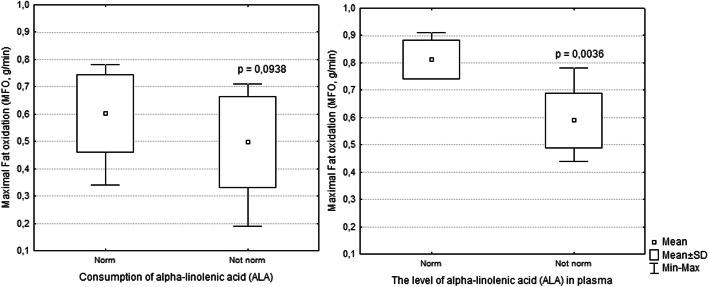
Maximum fat oxidation according to the level of ALA in the diet and plasma in skiers. *Note: Consumption of ALA – norm (n = 12) and not norm (n = 12); Level of ALA in plasma – norm (n = 4) and not norm (n = 20)*

The consumption of ALA in the diet in the total group was within the normal range (1.1–2.0 g/day), and the average was 1.3 ± 1.1 g/day. The consumption of ALA in the diet in 12 skiers was in the normal range at 2.1 ± 1.1 g/day. In individuals with a normal range of dietary intake of ALA, MFO was 0.6 g/min and ranged from 0.34 to 0.79 g/min. In the remaining 12 athletes, the consumption of ALA in the diet was below the recommended level (1.1–2.0 g/day) by – 0.5 ± 0.2 g/day (Fig. 2). For skiers who had a deficient intake of dietary ALA, MFO averaged 0.5 ± 0.2 g/day and ranged from 0.19 to 0.71 g/min (*p* = 0.0938). The correlation analysis showed that the consumption of ALA in the diet was associated with a higher MFO (r_s_ = 0.507, *p* = 0.011).

The average plasma ALA value in the total group was 0.3 ± 0.1 Mol %, which is lower than the recommended level (0.6 ± 0.1 Mol %). The plasma level of ALA was normal in four athletes and averaged 0.6 ± 0.2 Mol %, whereas the level in athletes with values below the recommendation averaged 0.2 ± 0.1 Mol %. In individuals with a normal plasma level of ALA, MFO was 0.81 g/min and ranged from 0.76 to 0.91 g/min. In individuals with plasma levels of ALA below the recommendation, MFO was 0.59 g/min and ranged from 0.44 to 0.79 g/min (*p* = 0.0036). The correlation analysis showed that the plasma level of ALA was also associated with a higher MFO (r_s_ = 0.460, *p* = 0.023).

**Fig. 3:**
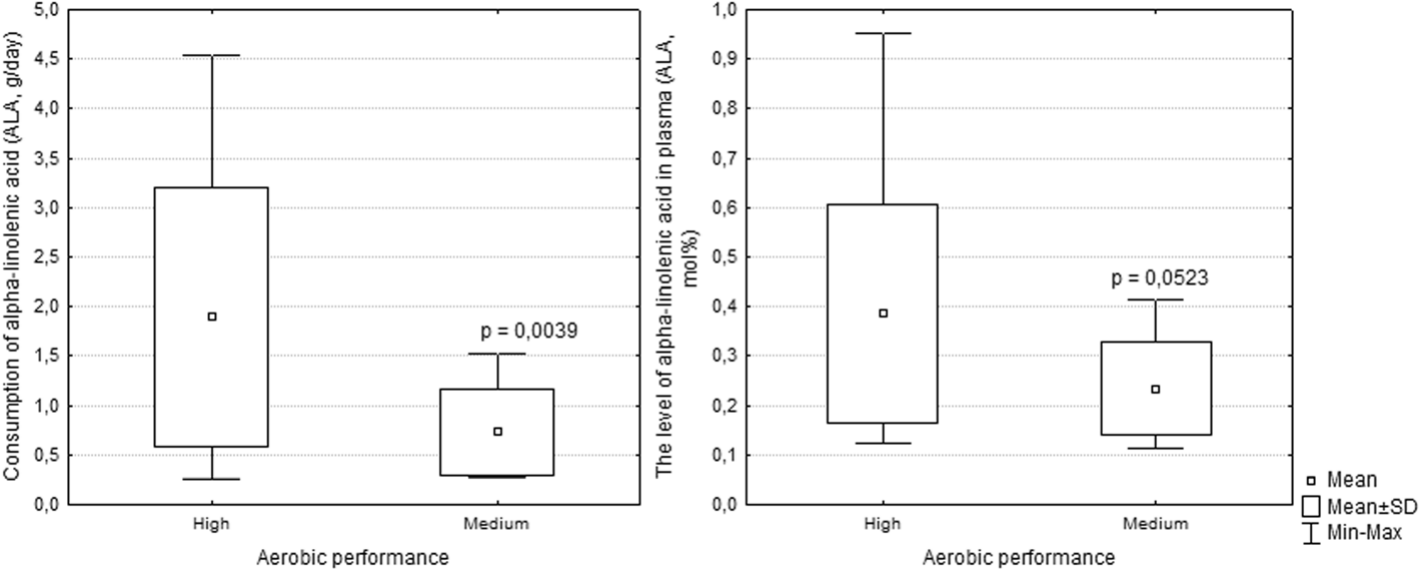
Aerobic performance according to the level of ALA in diet and in plasma in skiers. *Note: Consumption of ALA – high (n = 12) and medium (n = 12); Level of ALA in plasma – high (n = 13) and medium (n = 11)*

According to the literature, aerobic performance estimated through MFO is divided into 3 groups: low (MFO < 0.37 g/min), medium (MFO 0.37–0.69 g/min) and high (MFO > 0.69 g/min) [8]. As described above, adequate intake of ALA in the diet is associated with high aerobic performance among athletes (*p* = 0.0039). In individuals with high aerobic performance, the level of ALA in the diet was 1.9 g/day and ranged from 0.2 to 4.5 g/day. In individuals with an average aerobic performance, the level of ALA in the diet was 0.75 g/day and ranged from 0.2 to 1.5 g/day (Fig. 3).

Plasma levels of ALA were associated with aerobic performance (*p* = 0.052). In individuals with high aerobic performance levels, the average ALA level in plasma was 0.39 Mol % and ranged from 0.12 to 0.98 Mol %. Individuals with an average aerobic performance had an average plasma ALA level equal to 0.24 Mol % and ranged from 0.11 to 0.41 Mol % (recommended 0.6 ± 0.1 Mol %) [23].

## Discussion

In the present study, we investigated the link between the essential dietary and plasma ALA levels and aerobic performance (estimated via MFO) among skiers.

Physical activity of low and moderate intensity is accompanied by a 5- to 10-fold increase in FA oxidation relative to that at rest, and a maximum is reached at a load intensity at approximately 65% VO2max [11, 24]. The most likely mechanism of inhibition of FA oxidation at maximum power loads is low availability of free carnitine, a decrease in intracellular pH [11] and a decrease in carnitine palmitoyl transferase I activity and long-chain FA transport in mitochondria [25], which lead to a decrease in MFO [24–26]. Thus, well-trained athletes are believed to have an increased potential for fat utilization, which is accompanied by economical consumption of carbohydrates and reduces the development of fatigue [8].

There is evidence that the addition of the n-3 PUFA ALA and its transformation products eicosapentaenoic acid (EPA) and docosahexaenoic acid (DHA) promotes oxidation of FAs at rest and during exercise [18]. The significant role of n-3 PUFAs in optimizing physical performance has been established [14, 15], and ALA has been shown to improve the neuromuscular function of athletes [7], increase fat oxidation [8], and reduce inflammation [27]. To date, the biological effects of n-3 PUFAs have been largely attributed to improvements in mitochondrial bioenergy [28]; however, a key limiting step in the oxidation of FAs in skeletal muscles is the transport of lipids through the plasma membrane [18].

We found that the adequate consumption of ALA in the diet is associated with a higher value of MFO (Fig. 2), which is also shown in the literature [18, 29]. Chorner and colleagues have shown that the consumption of ALA for 12 weeks increases the content of n-3 PUFAs in the plasma membrane, the accumulation of plasma membrane FA translocase, the transport of FAs via sarcolemma, and the content of intramuscular triglycerides and MFO [18, 24]. The consumption of ALA in the diet increases the activity of carnitine palmitoyl transferase I and FA translocase (a key protein transporter of FAs), which in turn increases MFO in the mitochondria [18, 30]. ALA accounts for approximately 0.7% of the total FAs in adipose tissue and is actively mobilized during exercise, with ALA levels in plasma increasing by almost 6 times after prolonged intense exercise [17]. Given that more than half of the consumed ALA is converted into carbon dioxide to produce energy at a high rate, the participation of ALA in efficient energy supply is dominant relative to that of other n-3 PUFAs [30]. For all these reasons, ALA can function as an energy substrate during long-term training when carbohydrate reserves are depleted [17].

The lipid composition of skeletal muscle is sensitive to changes in the diet [31], especially when ALA or its conversion products EPA and DHA are potent activators of transcription factors, which are receptors activated by peroxisomal proliferators (PPARs) that regulate genes responsible for fat metabolism [32].

The addition of the essential FAs EPA and DHA in the diet increases the inclusion of n-3 PUFAs in the membrane and improves the sensitivity of mitochondria to ADP in muscle fibres [33], which contributes to the oxidation of fat and reduces the activity of carbohydrate-oxidizing enzymes (phosphorylase and pyruvate dehydrogenase) [34]. Taken together, these data suggest that n-3 PUFAs (ALA and its transformation products EPA and DHA) can promote fat oxidation through PPAR-dependent and PPAR-independent mechanisms [32].

High aerobic performance is associated with adequate dietary intake of EPA and DHA, which are both ALA conversion products (Fig. 3) and increase maximum oxygen consumption and physical performance [17, 18]. Dietary intake of EPA and DHA increases the efficiency of training at submaximal loads, which reflects the efficiency of oxygen consumption [14]. Since there is a close relationship between training efficiency and aerobic performance [18], intake of ALA in the diet increases aerobic performance.

## Limitations

This study has several limitations. First, taking blood to determine the ALA level at the peak of the bicycle ergometric load would be the most accurate method of determining high oxidation rates of this acid at the maximum load intensity (i.e., in the anaerobic work area). In addition, the subjectiveness in assessing nutrition among male athletes should be considered. Finally, the relatively small sample size (*n* = 24) may reduce the ability to draw strong and valid conclusions on the studied outcomes.

## Conclusions

In this study, we provided evidence that essential ALA in the diet plays an important role in increasing MFO and aerobic performance in skiers. Thus, this study indicates that the level of ALA should be increased in the diets of athletes because of the associated increase in the aerobic potential of the body via the use of essential FAs.

## Data Availability

All data are contained within the article. The datasets used and analysed during the current study are available from the corresponding author on reasonable request.

## Abbreviations

ALA: α-Linolenic acid
DHA: Docosahexaenoic acid
En%: Percentage of the total energy
EPA: Eicosapentaenoic acid
FAs: Fatty acids
FATmax: Intensity at which MFO occurred
HR: Heart rate
MFO: Maximal fat oxidation
PUFAs: Polyunsaturated fatty acids
QFat: Questionnaire of Fats
SAF RK: State Autonomous Institution of the Komi Republic
VO_2_max: Maximal oxygen uptake
